# Photon-counting CT angiography in a multi-trauma patient with bilateral pulmonary embolism: combined vascular mapping and quantitative iodine-density assessment prior to scalp reconstruction: case report

**DOI:** 10.3389/fmed.2026.1915043

**Published:** 2026-09-10

**Authors:** Kai Wei, Shuangxiang Lin

**Affiliations:** Department of Radiology, The Second Affiliated Hospital Zhejiang University School of Medicine, Hangzhou, China

**Keywords:** case report, computed tomography angiography, iodine mapping, multi-trauma, photon-counting CT, pulmonary embolism

## Abstract

**Background:** Photon-counting detector CT (PCCT) combines high-resolution anatomical and spectral imaging by directly counting individual x-ray photons and discriminating their energies. Its role in surgical planning in complex multi-trauma with concurrent pulmonary embolism remains undefined. Case Presentation: A 58-year-old previously healthy woman was entrapped by a cement mixer, sustaining traumatic scalp avulsion (20 × 20 cm) with exposed skull, depressed right parietal fractures, subdural and epidural hematoma, right 3rd–9th rib fractures, mid-shaft radius and ulna fractures with tendon and radial-nerve injury, and a left maxillary frontal-process fracture. After emergent craniotomy, debridement, open reduction and internal fixation of the forearm, and prolonged immobilization, bilateral soleal vein thrombosis and bilateral segmental pulmonary embolism developed. Before scalp reconstruction, PCCT angiography (NAEOTOM Alpha, Siemens) was performed with cinematic rendering and iodine-density mapping. Mean pulmonary iodine density was 1.82 mg/mL on the right versus 0.74 mg/mL on the left (right-to-left ratio 2.46:1), suggesting relatively preserved iodine distribution on the right and reduced iodine distribution on the left despite bilateral emboli on CTPA. A pedicled fasciocutaneous flap from the right anterolateral thigh was performed under tiered enoxaparin anticoagulation with anti-activated factor X (anti-Xa)–guided dose escalation. The flap survived completely, the donor site healed, and the patient was discharged on day 14 with stable wound coverage. At three-month follow-up she had no recurrent thromboembolism and recovered near-normal right upper-extremity function. Conclusion: PCCT angiography provided an arterial map for reconstructive planning and quantitative iodine maps in a single acquisition, supplementing CTPA with a quantitative surrogate of pulmonary blood-volume distribution. The novelty of this case is the integrated use of a single PCCT examination for vascular mapping and spectral pulmonary iodine assessment in a complex multi-trauma setting, not the demonstration of PCCT superiority over dual-energy CT. Whether this integrated approach changes outcomes compared with dual-energy CT protocols requires prospective comparison.

## Introduction

Photon-counting detector CT (PCCT) is a recently introduced clinical CT technology in which incident x-ray photons are converted directly into electrical pulses whose amplitude corresponds to photon energy (1, 2). Compared with conventional energy-integrating detector CT (EID-CT), PCCT offers smaller detector elements, lower electronic noise, and inherent energy discrimination (3–5). These properties allow PCCT to acquire high-resolution anatomical images alongside material-specific spectral data—including iodine-density maps, virtual monoenergetic images, and virtual non-contrast reconstructions—within a single acquisition and at a comparable or lower radiation dose than dual-energy EID-CT protocols (3–5). Iodine-density maps have previously been generated using dual-energy CT (DECT) techniques, including dual-source and rapid-kVp-switching systems, and have been applied to pulmonary perfusion assessment and pulmonary embolism evaluation (6). PCCT differs from DECT in that material decomposition is performed at the detector level on every photon, potentially offering improved energy separation, reduced electronic noise, and higher spatial resolution; however, the incremental diagnostic benefit of PCCT over contemporary dual-energy EID-CT for a given clinical task has not been established for most indications. In trauma, CT angiography (CTA) is the first-line modality for evaluating vascular injury, active hemorrhage, and organ damage because of its speed and ability to image the entire body within minutes (7, 8). Trauma-induced coagulopathy, prolonged immobilization, and venous injury also predispose these patients to venous thromboembolism (VTE), with reported rates of deep vein thrombosis exceeding 50% in high-risk trauma cohorts and pulmonary embolism rates of 4–22% in patients not receiving prophylaxis (9–11). Reconstructive surgery in patients on therapeutic anticoagulation carries a documented bleeding risk, but delaying surgery to complete a course of anticoagulation risks wound desiccation, infection, and further tissue loss. What is unique about this case is the combination of three features: (i) complex multi-trauma with extensive scalp avulsion requiring delayed reconstruction, (ii) concurrent bilateral pulmonary embolism that required careful perioperative anticoagulation, and (iii) the use of a single PCCT examination to provide both an arterial map for reconstructive planning and quantitative iodine maps for pulmonary blood-volume assessment in one contrast injection. Conventional EID-CT had already been performed earlier in the admission; the PCCT acquisition was added to provide additional spectral information that was not available from the prior EID-CT dataset.

### Patient information

A 58-year-old previously healthy woman with no known coagulopathy, prior thromboembolism, cardiovascular disease, diabetes, or chronic medication use was entrapped by a rotating cement mixer while working in mid-October 2024. She had no history of surgery, no known drug allergies, and no family history of bleeding or thromboembolic disorders. The right upper-extremity sleeve was pulled in first, followed by the head and chest, producing immediate severe pain, extensive scalp and right-arm skin avulsion, exposed skull, and exposed extensor tendons of the right hand. She remained conscious and oriented at the scene. At two regional hospitals she received wound dressing, tetanus prophylaxis, fluid resuscitation, and imaging that confirmed skull fracture, scalp avulsion, and right radius/ulna fractures, but persistent active bleeding and incomplete wound coverage led to transfer to our tertiary emergency center. There, additional imaging showed traumatic subdural hemorrhage and prompted emergent craniotomy, depressed-fracture elevation, and scalp debridement before transfer to the surgical intensive care unit.

### Clinical findings

At our center she was intubated and mechanically ventilated. Physical examination showed a 20 × 20 cm scalp defect with a 20 × 12 cm area of exposed calvarium, a 12 × 15 cm right axillary soft-tissue defect, exposed right-hand extensor tendons, multiple facial lacerations, and bilateral periorbital swelling with incomplete eyelid closure. Pupils were 2.5 mm and sluggishly reactive to light; cardiopulmonary and abdominal examinations were otherwise unremarkable. There was no clinical evidence of fat embolism, compartment syndrome, or rhabdomyolysis on serial examination. Admission laboratory values were: hemoglobin 90 g/L (anemia), platelets 57 × 10⁹/L (thrombocytopenia, possibly consumption-related), white blood cells 6.2 × 10⁹/L with neutrophil predominance (83.1%), C-reactive protein 37.1 mg/L (elevated), procalcitonin 1.78 ng/mL, interleukin-6 141.5 pg/mL, D-dimer 4560 µg/L FEU (markedly elevated), fibrinogen 5.08 g/L (upper normal range), prothrombin time activity 88%, and albumin 29.6 g/L (hypoalbuminemia). The combination of thrombocytopenia, hypercoagulability, marked D-dimer elevation, and prolonged immobility placed her at high VTE risk, with an estimated Padua score greater than 4 and an IMPROVE risk score supporting pharmacologic prophylaxis once bleeding risk allowed.

### Diagnostic assessment

**Figure 1:**
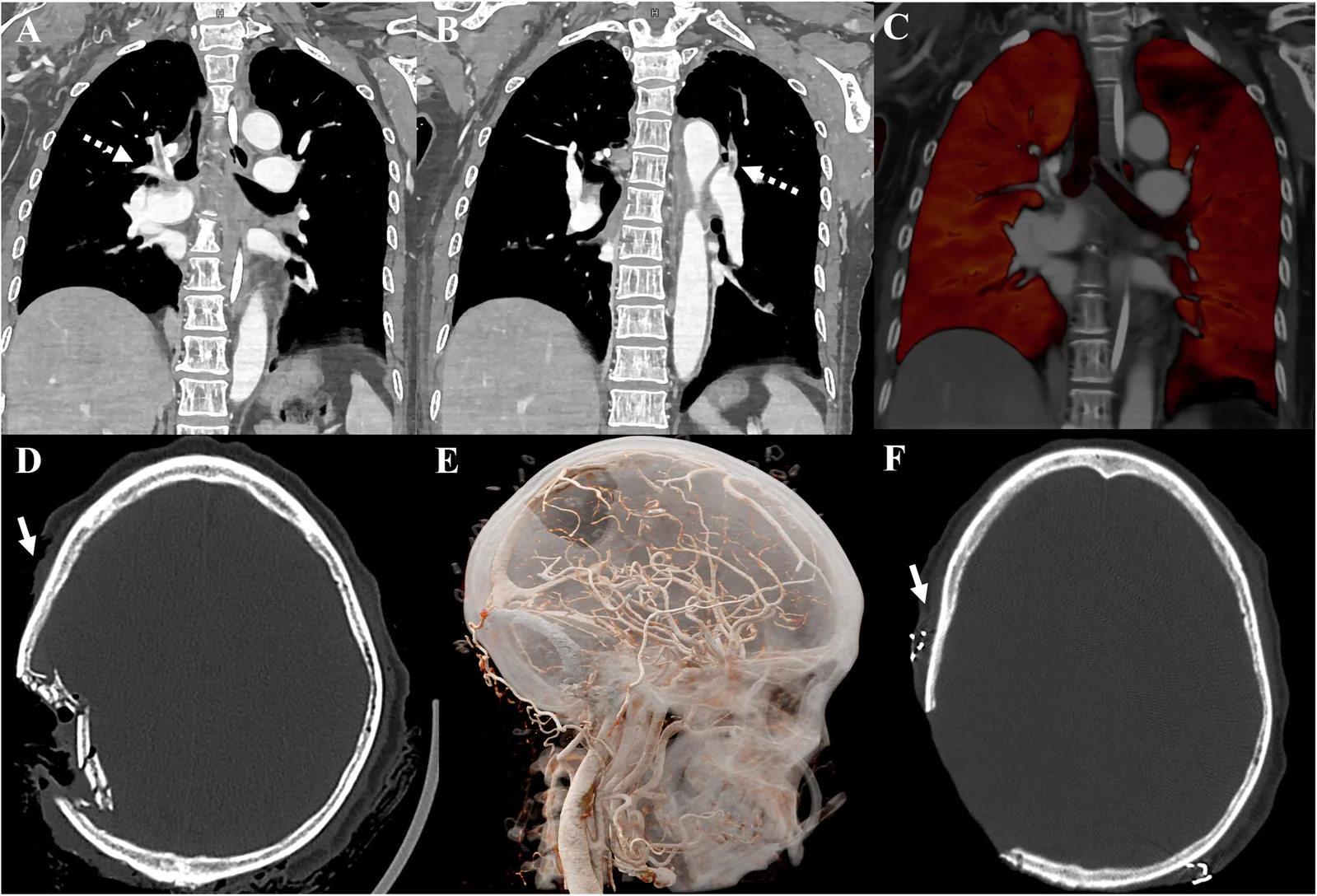
Pulmonary and cranial imaging of the trauma patient. **(A,B)** Coronal and axial CT pulmonary angiographic images demonstrate filling defects (white dashed arrows) in bilateral segmental and subsegmental pulmonary arteries, consistent with pulmonary embolism. **(C)** Iodine-density map in the coronal plane shows relatively preserved iodine distribution on the right lung (mean iodine concentration 1.82 mg/mL) and reduced iodine distribution on the left lung (mean 0.74 mg/mL); the color scale ranges from black (no iodine) to white (maximum iodine). Note that iodine density at a single arterial-phase acquisition is interpreted as a surrogate for pulmonary blood-volume distribution rather than as a direct measure of microvascular perfusion or gas exchange. **(D)** Axial image of the skull demonstrates the depressed right parietal fracture before surgery. **(E)** Cinematic volume-rendered image displays the course and distribution of the intracranial vessels after craniotomy for fracture elevation and epidural-hematoma evacuation. **(F)** Axial image of the head seven days after pedicled flap transplantation (white solid arrow) shows a viable, well-perfused flap.

**Figure 2:**
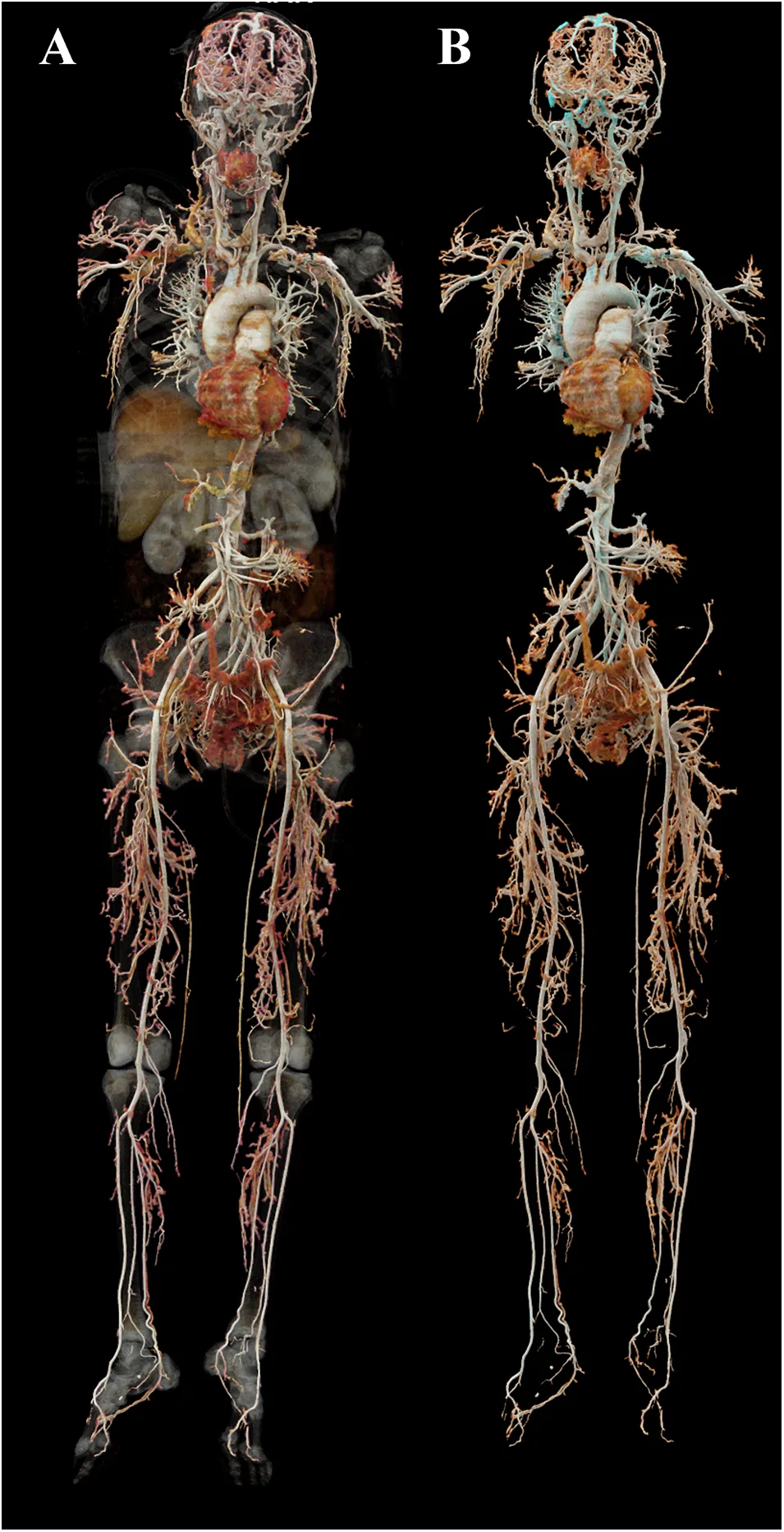
Whole-body vascular imaging of the trauma patient using photon-counting CT angiography reconstructed with cinematic rendering. The image provides a photorealistic three-dimensional overview of the cerebral, cervical, thoracic, abdominal, and lower-extremity arterial trees in a single view, allowing preoperative planning of the pedicled fasciocutaneous flap. **(A)** Anterior and **(B)** posterior views. Scale bar: 5 cm.

EID-CT (SOMATOM Definition Flash) on admission showed comminuted depressed right parietal fractures with extra-axial hematoma and pneumocephalus, a left maxillary frontal-process fracture, a focal left periorbital high-density focus (suspected foreign body), and right 3rd–6th and 8th rib fractures with possible incomplete fractures of the 7th and 9th ribs. The abdomen showed no acute findings. Transthoracic echocardiography on October 17 was unremarkable, with no right ventricular dilatation or pulmonary hypertension. Doppler ultrasound of the lower limbs on October 20 showed bilateral soleal and gastrocnemius intramuscular venous thrombosis with preserved compressibility of the deep veins. A follow-up lower-limb CTA on October 31 showed no arterial abnormality. Right upper-extremity radiographs confirmed mid-shaft fractures of the radius and ulna with an additional ulnar-side fracture of the distal radius. Because the patient now required definitive scalp reconstruction and donor-site selection depended on a precise vascular map, a whole-body PCCT angiogram (NAEOTOM Alpha) was performed on November 1, 2024 using a single iohexol injection (350 mg I/mL, 4 mL/s, 40 mL saline flush; bolus tracking in the descending aorta at 150 HU with a 7-s diagnostic delay). Acquisition parameters were: collimation 144 × 0.4 mm, rotation 0.25 s, pitch 0.9, tube voltage 120 kV, automatic tube-current modulation (reference mAs 180), and reconstructions at 0.6 mm and 3.0 mm slice thickness using a soft-tissue kernel (Qr36) and a bone kernel (Qr68) on a 512 × 512 matrix with iterative reconstruction (QIR-3, Siemens). CTDIvol was 8.4 mGy and DLP 622 mGy·cm, with an estimated effective dose of approximately 9.3 mSv using a chest conversion coefficient of 0.0145 mSv/mGy·cm. For spectral post-processing, two energy bins (20–50 keV and 50–80 keV) were used to generate iodine-density maps, virtual monoenergetic images at 40–80 keV, and virtual non-contrast reconstructions on a dedicated workstation (syngo.via VB60A, Siemens). The 20–50 keV and 50–80 keV bins were selected because the lower bin provides high iodine contrast sensitivity while the higher bin serves as a low-iodine reference, enabling two-material decomposition; this approach follows the vendor-recommended default for clinical PCCT iodine-mapping protocols on the NAEOTOM Alpha platform. Multi-planar reformation, maximum-intensity projection, cinematic rendering, and iodine-density maps were generated from the same acquisition. Iodine concentrations were measured by two independent readers (a radiology attending and a senior radiology resident, each with at least three years of experience in cardiovascular CT) who were blinded to each other's results. ROIs were drawn manually on axial iodine-density maps at three levels per lung (upper, middle, and lower zones, at the levels of the carina, the left atrium, and the inferior pulmonary veins, respectively). Each ROI was circular, approximately 1.0–1.5 cm^2^ in area, and was placed within the peripheral two-thirds of the lung parenchyma, deliberately excluding visible pulmonary vessels, airways, pleural surfaces, and areas of consolidation or atelectasis. Mean iodine density was 1.82 mg/mL (range 1.64–2.05) in the right lung and 0.74 mg/mL (range 0.58–0.91) in the left lung, giving a right-to-left ratio of 2.46:1. Inter-reader agreement was excellent (intraclass correlation coefficient 0.96; 95% CI 0.89–0.99). Reference values for normal pulmonary parenchyma on PCCT are approximately 1.5–2.5 mg/mL; values below 1.0 mg/mL have been proposed to indicate severely reduced iodine distribution (12, 13). The right-lung values fell within the reported normal range despite bilateral segmental pulmonary emboli on CTPA, whereas the left-lung values were below this threshold (Figure 1). Three-dimensional vessel-tracking identified a patent right anterolateral thigh perforator system suitable as the flap pedicle (Figure 2), and cinematic volume rendering reconstructed the entire arterial tree from the aortic arch to the pedal vessels (Figure 2; Supplementary Movie S1). The acquisition was completed in approximately 5 seconds of table travel.

### Therapeutic intervention

The patient underwent staged surgery during two admissions (October 16–29 and October 29–November 12, 2024). Table 1 summarizes the complete episode-of-care timeline. During the first admission, procedures included emergent depressed-fracture elevation and scalp debridement with left temporoparietal epidural-hematoma evacuation and vacuum-assisted closure (VAC) on the day of admission; right-hand necrotic-tissue debridement on POD 2 (broad-spectrum meropenem 0.5 g q8 h, POD 2–9); VAC exchange and scalp debridement on POD 5; open reduction and internal fixation of the right radius and ulna with simultaneous scalp-VAC exchange on POD 9; and definitive scalp and right axillary debridement with fasciocutaneous flap elevation and VAC coverage on POD 14. During the second admission, pedicled fasciocutaneous flap transplantation from the right anterolateral thigh with meshed split-thickness skin grafting was performed on November 4 (POD 19 from the first injury), followed by graft inspection and minor debridement on November 11. All procedures were under general anesthesia with endotracheal intubation. After the diagnosis of pulmonary embolism, a multidisciplinary team developed a tiered enoxaparin protocol aligned with EAST and CHEST recommendations (11, 14). Subcutaneous enoxaparin was started at 4000 IU q.d. [anti-activated factor X (anti-Xa) target 0.5–1.0 IU/mL] on October 24, 2024. After confirmation of bilateral segmental pulmonary embolism on PCCT, the dose was escalated to 4000 IU b.i.d. (anti-Xa target 1.0–1.5 IU/mL) on October 30. Enoxaparin was withheld 24 h before flap transplantation and resumed 12 h postoperatively at prophylactic dose for 72 h, then escalated to therapeutic dose once surgical bleeding risk was judged acceptable. Hemoglobin, anti-Xa activity, drain output, and flap perfusion were monitored throughout.

**Table 1:** Episode-of-care timeline.

Date (2024)	Event
Mid-October	Cement-mixer entrapment; emergent craniotomy, scalp debridement, and epidural-hematoma evacuation at regional hospital
Oct 16	Admission to our tertiary emergency center
Oct 18	Right-hand necrotic-tissue debridement; meropenem prophylaxis (POD 2–9)
Oct 20	Doppler ultrasound: bilateral soleal and gastrocnemius intramuscular venous thrombosis
Oct 21	Right-parietal wound VSD exchange and debridement
Oct 24	Enoxaparin 4000 IU s.c. q.d. started (anti-Xa 0.5–1.0 IU/mL)
Oct 25	ORIF of right radius and ulna; scalp VSD exchange
Oct 29	Second admission for scalp reconstruction; enoxaparin increased to 4000 IU b.i.d. (anti-Xa 1.0–1.5 IU/mL)
Oct 30	Definitive scalp and axillary debridement, fasciocutaneous flap elevation, VAC coverage
Oct 31	Lower-limb CTA: no arterial abnormality
Nov 1	Comprehensive PCCT angiography with cinematic rendering and iodine-density mapping
Nov 4	Pedicled fasciocutaneous flap from right anterolateral thigh with meshed split-thickness skin grafting
Nov 11	Skin-graft inspection and minor debridement
Nov 12	Discharge; transition to rivaroxaban 20 mg daily for ≥3 months
Feb 2025	Three-month follow-up: stable wound coverage, no recurrent VTE, near-normal right upper-extremity ROM

### Follow-up and outcomes

The flap survived completely (100%) without congestion, ischemia, or partial necrosis, with a positive handheld Doppler signal at the flap edge on every postoperative day. The donor site achieved 100% re-epithelialization within two weeks. At discharge, active right shoulder flexion measured 90° and forearm pronation–supination 60°/70° (uninjured left 170°/80°/85°). At three-month follow-up, the patient had stable wound coverage, active right shoulder flexion improved to 160°, forearm pronation–supination 75°/80°, and no recurrent VTE on Doppler ultrasound. She had returned to her previous activities of daily living.

## Discussion

This case describes an integrated preoperative imaging strategy in a complex multi-trauma patient, in which PCCT angiography contributed three elements within a single acquisition: a high-resolution arterial map for reconstructive planning, cinematic volume-rendered visualization of the systemic arterial tree, and quantitative iodine maps of pulmonary iodine distribution. These data were considered alongside the broader clinical assessment, including laboratory results, echocardiography, and Doppler ultrasound, when formulating the perioperative plan. The discussion below acknowledges the limitations of attributing clinical decisions to any single imaging modality and compares the present approach with dual-energy CT alternatives. Iodine maps as a surrogate for pulmonary blood-volume distribution. Iodine-density maps derived from PCCT (or DECT) reflect the local concentration of iodinated contrast within the lung parenchyma during a single arterial-phase acquisition. They are generally interpreted as a surrogate for pulmonary blood-volume distribution rather than as a direct measurement of true microvascular perfusion, gas exchange, or ventilation/perfusion matching (6, 13, 15, 16). In this case, normal right-lung iodine density in the presence of bilateral segmental emboli on CTPA was interpreted as preserved iodine distribution on the right and as a finding inconsistent with extensive right-sided microvascular occlusion, which contributed to the multidisciplinary impression that the right lung was likely to tolerate the perioperative period under therapeutic anticoagulation. We did not perform nuclear perfusion scintigraphy or dual-input CT perfusion; therefore, the relationship between iodine density and true regional perfusion or gas exchange in this patient was not directly verified. The asymmetry between right and left lung iodine density should also be interpreted cautiously, because iodine concentration can be influenced by cardiac output, contrast-bolus timing, gravity-dependent lung zones, and the presence of adjacent consolidation or atelectasis. Incremental value of PCCT compared with dual-energy CT. Iodine maps, virtual monoenergetic images, and material decomposition are well-established features of contemporary dual-energy EID-CT systems, and dual-energy approaches have previously been used for pulmonary embolism and trauma imaging (6, 16). In this case, conventional EID-CT had already been performed on admission (SOMATOM Definition Flash, dual-source), but a dedicated dual-energy pulmonary acquisition with iodine mapping was not included at that time. The PCCT study was therefore not directly compared with a contemporaneous DECT dataset in the same patient. The specific contributions that PCCT offered in this examination included: (i) higher spatial resolution for depicting small perforator vessels used in flap planning (1, 17, 18), (ii) the ability to combine a whole-body arterial CTA with iodine maps in a single acquisition on a single contrast injection, and (iii) reduction of beam-hardening artifact around the metallic implants used in the prior right forearm fixation. Whether these features would translate into measurable clinical benefit—shorter operative time, fewer complications, lower contrast volume, or reduced re-imaging—compared with an equivalent dual-energy EID-CT protocol cannot be determined from a single case and warrants prospective intra-individual comparison. The reported dose in this study (CTDIvol 8.4 mGy; effective dose ∼9.3 mSv) is comparable to published dual-energy and PCCT trauma protocols (19, 20). Multidisciplinary context. The decision to proceed with reconstructive surgery under therapeutic anticoagulation was made by a multidisciplinary team (emergency surgery, respiratory medicine, plastic surgery, rehabilitation, and pharmacy) and was based on the integrated clinical picture, including hemodynamic stability, normal echocardiographic findings, laboratory values, Doppler ultrasound results, and the imaging data. PCCT angiography provided additional quantitative information on pulmonary iodine distribution and a detailed vascular map, but the surgical decision should not be attributed to PCCT alone. Other management options—such as delayed reconstruction after a full course of anticoagulation or placement of a retrievable inferior vena cava filter—would also have been reasonable in similar circumstances and were explicitly considered by the team. Limitations. The advantage of PCCT over EID-CT in this scenario is qualitative rather than outcome-based; no randomized or intra-individual comparison was performed, and the patient had not undergone a contemporaneous DECT acquisition for direct head-to-head comparison. The reported right-left iodine ratio should not be interpreted as evidence of true perfusion asymmetry without corroborating functional imaging. Iodine-density values are device- and reconstruction-dependent, with limited inter-vendor standardization (21); our cut-off of approximately 1.0 mg/mL to indicate severely reduced iodine distribution was derived from recent PCCT literature and has not been prospectively validated, and although two readers performed the measurements in this case, the broader inter-reader reproducibility of pulmonary iodine mapping on PCCT has not been characterized. The lower-lung ROIs may have been influenced by gravity-dependent atelectasis, and bolus-timing differences between zones cannot be excluded. The tiered anticoagulation protocol was based on extrapolation from VTE prophylaxis and treatment guidelines (11, 22) rather than on validated recommendations specific to trauma patients with concurrent PE who require reconstructive surgery. Selection bias is acknowledged because PCCT was performed specifically for donor-site planning; broader utility in less complex trauma cannot be inferred from a single case. Cinematic rendering requires dedicated post-processing expertise, and the learning curve for interpreting iodine maps in the trauma setting has not been characterized. Relation to prior literature. PCCT has been evaluated in cardiovascular, thoracic, and musculoskeletal imaging (12, 17, 23–25), and a recent intra-individual study showed superior infrapopliteal CTA on PCCT versus EID-CT (26). Iodine maps have been reported to aid pulmonary embolism assessment (27), and dose reduction with PCCT has been documented in whole-body and dedicated protocols (28, 29). Dual-energy EID-CT has previously been used to derive iodine maps in pulmonary embolism and trauma (6). This case adds to those findings by describing the integrated use of a single PCCT examination for systemic arterial mapping and spectral pulmonary iodine assessment in a complex multi-trauma setting, and by reporting the specific acquisition parameters (energy-bin selection, ROI methodology) used. Implications. For clinicians managing complex multi-trauma with concurrent VTE who require reconstructive surgery, the present experience suggests that iodine-density maps may complement CTPA by providing a quantitative surrogate of pulmonary iodine distribution, which can inform multidisciplinary risk stratification. The novelty of this case is the combined use of a single PCCT acquisition for vascular mapping and pulmonary iodine assessment; whether this integrated approach changes outcomes compared with contemporary dual-energy CT protocols remains to be determined in prospective comparative studies.

## Data Availability

The original contributions presented in the study are included in the article/Supplementary Material, further inquiries can be directed to the corresponding author.
